# Metacognitions in Patients With Frequent Mental Disorders After Diagnosis of Pulmonary Arterial Hypertension

**DOI:** 10.3389/fpsyt.2022.812812

**Published:** 2022-04-14

**Authors:** Flora Caldarone, Philippa Gebhardt, Marius M. Hoeper, Karen M. Olsson, Jan Fuge, Da-Hee Park, Tanja Meltendorf, Jan C. Kamp, Britta Stapel, Manuel J. Richter, Henning Gall, Hossein A. Ghofrani, Kai G. Kahl, Ivo Heitland

**Affiliations:** ^1^Department of Psychiatry, Social Psychiatry and Psychotherapy, Hannover Medical School, Hannover, Germany; ^2^Department of Respiratory Medicine, Hannover Medical School, Member of the German Center for Lung Research (DZL/BREATH), Hannover, Germany; ^3^Biomedical Research in Endstage and Obstructive Lung Disease Hannover (BREATH), Hannover, Germany; ^4^Department of Internal Medicine, German Center for Lung Research (DZL), Justus Liebig University Giessen, Universities of Giessen and Marburg Lung Center (UGMLC), Giessen, Germany; ^5^Department of Pneumology, German Center for Lung Research (DZL), Kerckhoff Heart, Rheuma and Thoracic Center, Universities of Giessen and Marburg Lung Center, Bad Nauheim, Germany

**Keywords:** pulmonary arterial hypertension (PAH), pulmonary hypertension, adjustment disorder, depressive disorder, panic disorder, metacognitions, psychocardiology

## Abstract

**Background:**

The prevalence of mental disorders, particularly adjustment disorder (AD), major depressive disorder (MDD) and panic disorder (PD) is increased in patients with pulmonary arterial hypertension (PAH). However, it is unclear which pathogenic mechanisms determine their development and could therefore be targeted in prevention or therapeutic interventions. Here, we assessed metacognitions in a sample of PAH patients with and without MDD and PD. Moreover, we reconstructed the course of mental illnesses following the PAH diagnosis.

**Methods:**

Two hundred seventeen PAH patients were included in this cross-sectional study. The prevalence of AD was assessed retrospectively using DSM-V criteria. Current mental disorders were assessed using the structured clinical interview for DSM-V. Additionally, metacognitive beliefs and processes were assessed using established questionnaires (MCQ-30, AnTI).

**Results:**

Patients with an AD consecutive to the PAH diagnosis more frequently developed MDD (37.5 vs. 13.9%, *p* < 0.001) and PD (26.3 vs. 8.8%, *p* = 0.001) later on compared to PAH patients without a former AD. Moreover, patients with current MDD/PD displayed more dysfunctional metacognitions than those without current MDD/PD (*p* < 0.001). Patients with current MDD/PD in the context of former AD had more dysfunctional metacognitive worries and beliefs compared to patients with current MDD/PD without former AD (*p* = 0.009).

**Conclusion:**

Our results suggest that in the context of PAH, dysfunctional metacognitions are associated with MDD and PD. Therefore, a metacognitive approach to treat and prevent those mental illnesses seems promising and should be investigated in future studies.

## Introduction

Pulmonary arterial hypertension (PAH) is a rare cardiopulmonary disease characterized by increased pressure in the pulmonary arteries and increased pulmonary vascular resistance (1). Clinically, it often manifests as impaired physical capability due to dyspnea at exertion, fatigue, weakness as well as angina, syncope, and edema (1). Although there have been tremendous advances in therapy in the past years which have led to improved survival rates and disease control, PAH remains progressive, to date incurable and may lead to right heart failure and death (2, 3).

The impact of PAH extends beyond impaired physical capabilities. Research shows that PAH is associated with limitations regarding work and employment, finances, relationships, and emotional wellbeing (4). For many patients, the emotional distress caused by the disease and its associated limitations persists and mental illness arises. Previous studies showed that over one third of PAH patients develop symptoms of mental disorders (5–8). Olsson et al. (9) systematically investigated the prevalence of mental disorders in PAH patients with structured face-to-face interviews and found that current or past adjustment disorder (AD), current major depressive disorder (MDD) and panic disorder (PD) were the most prominent illnesses. Little research focused on the actual psychological mechanisms that lead to the increased risk of developing a mental disorders being a PAH patient. The current ESC/ERS guidelines for pulmonary hypertension take the psychological impact and prevalence of mental disorders into account and include a recommendation for psychosocial support (1). In daily clinical practice, however, only a small amount of patients receive adequate psychological treatment (5, 6). To gain an understanding of mental disorders in the context of PAH and to develop an evidence-based and tailored treatment, the underlying mechanisms of the development and maintenance of mental disorders have to be investigated.

One approach to transdiagnostically address mental illnesses, also in the context of severe physical illnesses, is the metacognitive theory by Wells (10), which is based on the Self-Regulatory Executive Function model (S-REF) (11). Metacognitive theory postulates that metacognitions, i.e., the cognitive processes that are involved in interpreting, monitoring and controlling ones' cognition, play a decisive role in the development and maintenance of mental disorders (12). The Cognitive Attentional Syndrome (CAS), which is characterized by inflexible attention, worry and rumination, threat monitoring and dysfunctional coping, leads to the inability to process normal emotional distress and results in its maintenance (10). So-called metacognitive beliefs, i.e., beliefs about cognitive processes, are assumed to have an important impact on the individual thinking styles (11). The beliefs can be separated into five different domains: 1) Positive beliefs about worry, 2) negative beliefs about worry, 3) beliefs about cognitive competence, 4) beliefs about the need to control thoughts, and 5) cognitive self-consciousness (13–15).

A number of studies examined the role of metacognitions in psychopathology: Metacognitions are associated with major depressive disorder, generalized anxiety disorder, obsessive-compulsive disorder (16), shyness and social anxiety (17, 18), eating disorders (19), psychosis (20), PTSD (21), addictive behaviors (22), and also risk factors for psychopathology, for example adverse childhood experiences (23). Research indicates that the degree of metacognitions differs depending on the course of the mental disorder. For example, Halvorsen et al. (24) found that there are higher levels of metacognitions in currently depressed patients than in formerly or never depressed patients. Research regarding the temporal sequence also showed that metacognitions predict later psychiatric symptoms, for example of depression and anxiety (25–27). Regarding somatic illnesses, two recent systematic reviews have shown that dysfunctional metacognitions are associated with psychiatric symptoms and poor life quality in patients with chronic physical illnesses (27, 28). Most studies investigating metacognitions in physical illnesses appear to focus on cancer and neurological disorders. Winter et al. (29) performed a case study using a metacognitive approach for AD in a PAH patient, which supports the assumed role of metacognitions in PAH patients as well: After a short treatment targeting dysfunctional metacognitions the psychiatric disorder was in remission. To our knowledge, no study has systematically assessed metacognitions and their associations with mental illnesses in patients with cardiopulmonary disorders. Based on the research on metacognitions in mental disorders in general and specifically in chronic physical illnesses, it seems plausible that metacognitions play a role in psychiatric symptoms in patients with PAH as well.

The aim of this study is to explore the underlying mechanisms of mental disorders in patients with PAH in a theory-driven manner. We aim to assess metacognitions in PAH patients and investigate whether there is a difference in metacognitions in patients who do not develop one of the most common mental disorders after the PAH diagnosis and those who do. Additionally, we are interested in reconstructing the course of mental disorders after the diagnosis and in the metacognitions associated with it.

## Methods

### Participants and Procedure

Patients were recruited from two German pulmonary hypertension referral centers (Hannover Medical School and University of Gießen and Marburg). The local institutional review boards of the participating universities approved all study procedures (Nr. 8540_BO_K_2019 for Hannover and Nr. 21119 for Gießen and Marburg). Inclusion criteria were: 1. confirmed diagnosis of PAH (1), 2. minimum age of 18 years and 3. ability to complete the questionnaires and the clinical interview in German. Patients meeting the inclusion criteria were either identified from a database and invited to participate via mail or recruited during their ambulatory visits. After providing informed written consent, patients completed a set of questionnaires and the structured clinical interview for DSM-V. We contacted 327 patients, 217 of them fulfilled the inclusion criteria and completed the questionnaires and the clinical interview. The final study group consisted of 155 females (71%), with an average age of 55 ± 14.58 years.

### Measures

#### Metacognitions Questionnaire-30

The MCQ-30 (13) is a self-report scale that measures beliefs about worry, monitoring tendencies and thinking itself. As the short-form of the MCQ (14), the five-factor structure of the original form was confirmed for the MCQ-30 as well. The resulting subscales are positive worry beliefs, beliefs about uncontrollability and danger of thoughts, beliefs about cognitive competence, beliefs about need to control thoughts, and cognitive self-consciousness. The 30 items are answered on a 4-point Likert scale from 1 (“do not agree”) to 4 (“agree very much”). The instrument has good internal consistency, convergent and construct validity and acceptable to good test–retest reliability (13).

#### Anxious Thoughts Inventory

The AnTI is a multidimensional self-report measure of worry (30). It consists of three subscales: social worry, health worry and meta-worry (worry about worry), which are factorially reliable. The 22 items are rated using a 4-point Likert scale from 1 (“almost never”) to 4 (“almost always”). The instrument has good psychometric properties (alpha coefficients of the subscales range between 0.75 and 0.84, 6-week test-retest reliability shows correlations between 0.76 and 0.84) (30).

#### Structured Clinical Interview for DSM-V

The SCID-V was used to examine currently present mental illnesses according to the Diagnostic and Statistical Manual of Mental Disorders, Fifth Edition (DSM-5) (31). Diagnoses during the last 4 weeks were evaluated. Additionally, patients were asked whether their current symptoms were independent from, worsened or caused by the PAH diagnosis. The SCID-V was also used for a retrospective appraisal to assess the prevalence of adjustment disorders within 3 months after the PAH diagnosis.

### Statistical Analysis

IBM SPSS Statistics 26.0 (IBM Corp, Armonk, NY, USA) was used for data analysis. Prevalence of current mental disorders in patients with and without former AD following PAH diagnosis were compared using Chi^2^-tests. To examine the prevalence of dysfunctional metacognitions in individuals with or without MDD/PD diagnosis, multivariate analyses were performed with questionnaire scores as dependent variables and current psychiatric diagnosis (MDD and PD) as well as former AD as independent variables. To determine specific group differences, *post-hoc* tests with Bonferroni correction were used. Patients who reported that their current mental illness was not connected to the somatic diagnosis were excluded. *P*-values below 0.05 were considered statistically significant.

## Results

### Descriptive Statistics

Subject characteristics are shown in Table 1.

**Table 1:** Participant characteristics.

	**Study sample (*n* = 217)**
Age (years)	55 (±14.58)
Female sex (%)	155 (71%)
Time since PAH diagnosis (years)	8.11 (±7.08)
Current MDD	50 (23.0%)
MDD caused or worsened by PAH diagnosis^a^	49 (22.6%)
Current PD	33 (15.2%)
PD caused or worsened by PAH diagnosis^a^	33 (15.2%)
AD following PAH diagnosis	80 (36.9%)
Current psychotherapy	22 (10.1%)
Current psychopharmacological treatment	25 (11.5%)

*Continuous variables are reported as mean and SD, categorical variables as n and percent*.

a *Reported by patients*.

### Prevalence of MDD and PD Considering Former AD

Patients who reported an AD following the diagnosis of PAH showed significantly higher rates of current MDD, compared to patients without AD after diagnosis of PAH [χ^2^_(1)_ = 16.13, *p* < 0.001, φ = 0.24]. PD also was more prevalent in patients with former AD following PAH compared to those without former AD [χ^2^_(1)_ = 11.93, *p* = 0.001, φ = 0.27]. See Table 2 for full data.

**Table 2:** Prevalence of MDD and PD in patients with and without former AD.

	**AD after PAH diagnosis (*****n*** **= 80)**		**no AD after PAH diagnosis (*****n*** **= 137)**		***p*-value**	**φ**
	***n***	**%**	***n***	**%**		
Current MDD	30	37.5	19	13.9	<0.001	0.24
Current PD	21	26.3	12	8.8	0.001	0.27

*AD, adjustment disorder; MDD, major depressive disorder; PD, panic disorder. The p-values and the effect size φ of the Chi^2^-test are reported*.

### Metacognitions

To examine dysfunctional metacognitions, a MANOVA with AnTI and MCQ-30 scores as dependent variables and current MDD/PD and former AD as independent variables was performed.

There was a significant multivariate main effect of current MDD/PD on the combined dependent variables [Wilks's Λ = 0.68, *F*_(8,206)_ = 11.88, *p* < 0.001, partial η^2^ = 0.32]. The multivariate main effect of former AD on the combined dependent variables was not significant [Wilks's Λ = 0.94, *F*_(8,206)_ = 1.78, *p* = 0.079, partial η^2^ = 0.07]. The multivariate interaction effect remained significant [Wilks's Λ = 0.91, *F*_(8,206)_ = 2.71, *p* = 0.07, partial η^2^ = 0.10].

**Figure 1:**
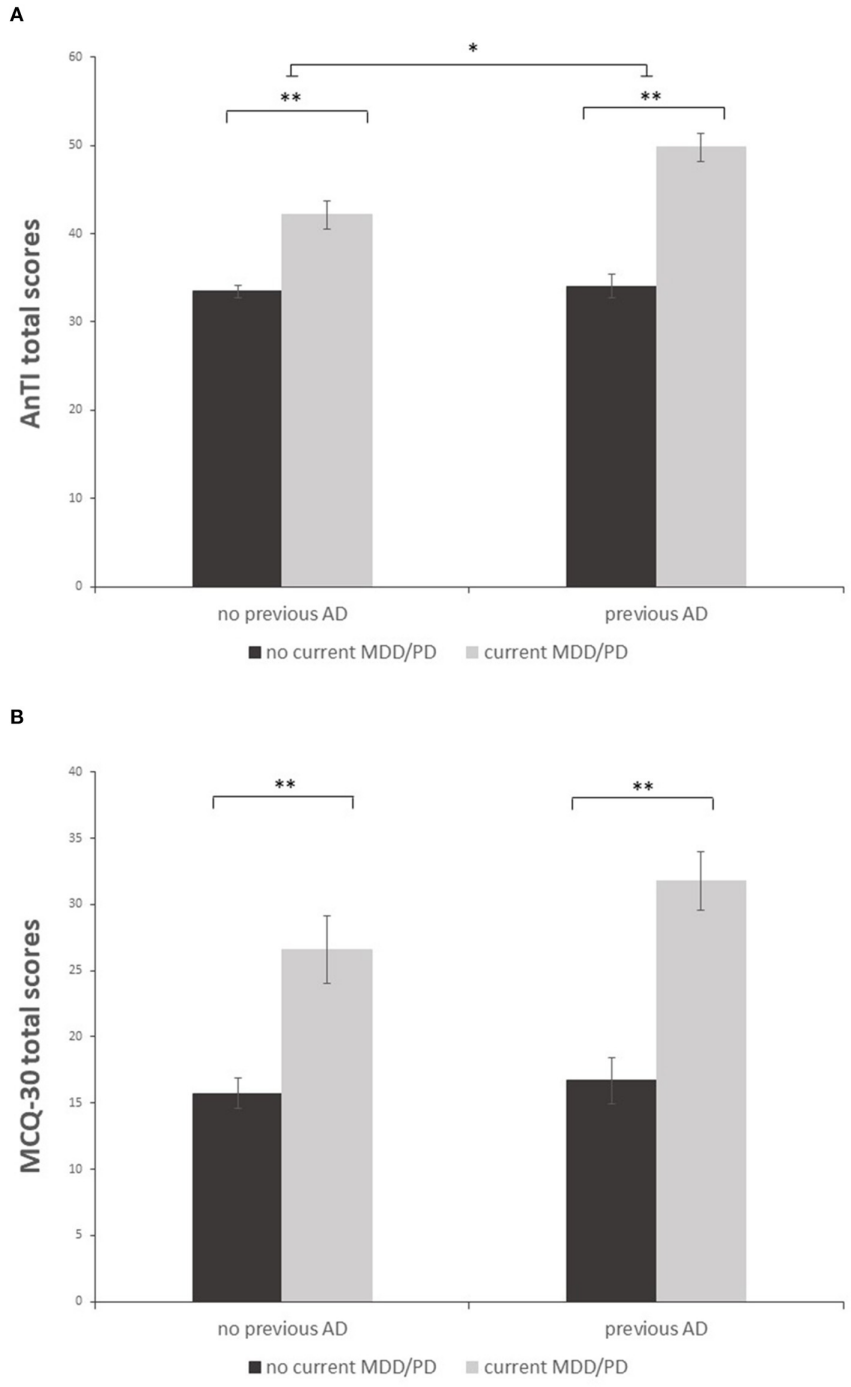
Mean sum scores of the AnTI **(A)** and MCQ-30 **(B)** in patients with and without both previous AD and current MDD/PD. Depicted are two significant main effects for the factors current MDD/PD and previous AD **(A)** and one significant main effect for the factor current MDD/PD **(B)**. Error bar indicate ±1 standard error of the mean. **p < 0.001; *p < 0.01.

Univariate analyses revealed a significant main effect showing that patients with current MDD/PD displayed more dysfunctional metacognitions than currently mentally healthy individuals for both sum scores of the questionnaires [AnTI: *F*_(1,213)_ = 84.03, *p* < 0.001, partial η^2^ = 0.28; MCQ-30: *F*_(1,213)_ = 44.71, *p* < 0.001, partial η^2^ = 0.17], see Figure 1. Following univariate analyses of the subscales of both questionnaires showed that patients with current MDD/PD reported higher levels of metacognitions than those without current MDD/PD regarding all subscales of the AnTI and the MCQ-30. Albeit, effect sizes of the respective subscales differed in magnitude. For Details, see Table 3.

**Table 3:** Mean scores of the AnTI and MCQ-30 and their subscales in patients with and without current MDD/PD.

	**No current MDD/PD,*M (SD)***	**Current MDD/PD,*M (SD)***	***F*(*df* 1, 213)**	***p***	**Partial η^2^**
AnTI sum score	33.60 (8.17)	46.69 (9.80)	84.03	<0.001	0.28
AnTI: social worry	13.16 (3.47)	18.50 (5.19)	62.27	<0.001	0.23
AnTI: health worry	10.28 (2.74)	13.53 (3.81)	41.00	<0.001	0.16
AnTI: meta-worry	10.16 (3.20)	14.66 (3.91)	62.81	<0.001	0.23
MCQ-30 sum score	15.99 (11.93)	29.68 (13.32)	*44.71*	<0.001	*0.17*
MCQ-30: positive worry beliefs	2.38 (2.73)	4.69 (3.40)	22.04	<0.001	0.09
MCQ-30: beliefs about uncontrollability and danger of thoughts	2.95 (3.20)	7.55 (3.84)	67.58	<0.001	0.24
MCQ-30: beliefs about cognitive competence	2.85 (3.01)	4.29 (3.94)	6.01	0.015	0.02
MCQ-30: beliefs about need to control thoughts	3.48 (3.16)	5.63 (3.50)	17.68	<0.001	0.08
MCQ-30: cognitive self-consciousness	4.34 (3.37)	7.52 (3.41)	32.80	<0.001	0.13

*AnTI, Anxious Thoughts Inventory; MCQ-30, Metacognitions Questionnaire-30; MDD, major depressive disorder; PD, panic disorder. Mean and standard deviation of the questionnaire scores and subscores, F-statistics, degrees of freedom, p-values and effect size partial η^2^ are reported*.

For the AnTI, there was a significant main effect showing that patients with former AD displayed more dysfunctional metacognitions than those without former AD [*F*_(1,213)_ = 9.66, *p* = 0.002, partial η^2^ = 0.04]. For the MCQ-30, this main effect was not significant [*F*_(1,213)_ = 2.50, *p* = 0.115, partial η^2^ = 0.01]. For the AnTI, there was a significant effect regarding the interaction of current MDD/PD and former AD [*F*_(1,213)_ = 6.99, *p* = 0.009, partial η^2^ = 0.03]. For the MCQ-30, this interaction effect was not significant [*F*_(1,213)_ = 1.16, *p* = 0.282, partial η^2^ = 0.01]. A *post-hoc* t-test with Bonferroni correction was performed. The *post-hoc* test remained significant [*t*_(60)_ = −3.25, *p* = 0.002], showing that patients with current MDD/PD and former AD (*M* = 49.78, *SD* = 9.85) had more dysfunctional metacognitions than patients with current MDD/PD without former AD (*M* = 42.12, *SD* = 7.88).

## Discussion

The current study revealed an association between an AD after the diagnosis of PAH and consecutive mood and anxiety disorders. Elevated levels of dysfunctional metacognitions were shown amongst PAH patients with mental disorders, varying depending on the course of the mental disorder.

Patients who suffered from AD after the somatic diagnosis frequently developed MDD and PD. This finding is consistent with research regarding the long-term prognosis of AD, which considers AD as a gateway to a severe psychiatric illness (32). This provides further insights into the psychological process that may be induced by living with PAH. It also stresses the importance of early screening for possible psychological distress after the somatic diagnosis. A screening could be done via a short questionnaire issued by the attending physician, after an acute somatic event or the diagnosis itself. As a screening tool, the Hospital Anxiety and Depression Scale could be used, which's utility was shown for PAH patients (9). Olsson et al. (9) also suggested developing a specific questionnaire for the symptoms of AD. An affirmative screening would path the way to more in depth diagnostics and enable a timely multidisciplinary approach including psychological treatment.

Patients with current MDD/PD reported more dysfunctional metacognitions than those without current mental health issues. This result adds to the current state of knowledge regarding metacognitions in patients with somatic disorders (27, 28), demonstrating that they are also associated with mental disorders in the context of PAH. Findings show a heightened rate of dysfunctional metacognitions in general, however, some facets appear more salient. Especially all three facets of the AnTI (social, health and meta-worry) and beliefs about the uncontrollability and danger of thoughts are most prominent in PAH patients with current MDD/PD. Additionally, findings suggest that metacognitions vary across the course of mental disorders. This is shown by the different amount of metacognitions in currently mentally ill patients depending on a former AD. Possibly, dysfunctional metacognitions are a pathogenic mechanism for the risk AD states for the development of a later MDD/PD: If a person develops an AD and the correlating metacognitions, these metacognitions might persist and even increase over time, leading to a higher chance of a MDD or PD with a high amount of dysfunctional metacognitions. A longitudinal study assessing metacognitions and their development over the course of these mental disorders is needed to investigate this assumption. Furthermore, the severity of the later on developed mental disorder should be assessed to see whether the former AD and more metacognitions lead to a higher symptom load. Contrary to our expectations, a higher level of metacognitions in MDD/PD patients with former AD compared to those without former AD only appeared in the AnTI, not in the MCQ-30. Both questionnaires are self-report measures assessing metacognitions. The MCQ-30 covers a broader spectrum of metacognitive processes and beliefs (13) while the AnTI focuses on different dimensions of worries (30). A possible reason for the discrepancy described above might be that the target group consisted of patients with a severe somatic illness with a bad prognosis. It seems plausible that in this specific group, especially in those who struggle with adjusting to the diagnosis, continuous health worries are most prominent. As the AnTI focuses more on worries about physical health than the MCQ-30, this stronger representation could be an explanation for the difference.

The findings have clinical implications: To prevent or treat the investigated mental disorders in the context of PAH, targeting dysfunctional metacognitive beliefs and processes seems promising. In metacognitive therapy (MCT) (12), techniques like attention training and detached mindfulness are used to modify metacognitions, establish a more flexible thinking style and thus reduce psychological distress. The effectiveness of MCT has been shown, especially for anxiety and depression (33). The current findings also contribute to the preliminary evidence that targeting dysfunctional metacognitions through MCT seems promising in this specific patient group in the context of PAH (29).

Our study has limitations: The cross-sectional design does not allow causal interpretation and limits conclusions about the course of the psychiatric illnesses and the underlying mechanisms. We decided to include the diagnosis of adjustment disorder in our study, however, there is an ongoing controversy regarding its validity. The retrograde assessment of AD bears a risk of recall bias. A longitudinal study should be conducted to examine the assumed positive correlation between severity of psychiatric symptoms and of metacognitions.

Future studies will have to continue to explore the proposed assumption that in the PAH patient group health worries might be more prominent than other metacognitions. Furthermore, it is necessary to address the role of metacognitions and their variation across the course of the mental disorders in more detail. The development of a tailored treatment based on the findings regarding metacognitions could be promising. That treatment could address the persevering health worry and other prominent metacognitions by using the MCT.

In conclusion, metacognitions are associated with MDD and PD in patients with PAH. That opens up potential for psychological support based on MCT: After a PAH diagnosis, a preventive metacognitive approach could be used to avoid the development of AD and later on MDD/PD and metacognitive therapeutic approaches could be used to treat AD, MDD and PD.

## Data Availability Statement

The raw data supporting the conclusions of this article will be made available by the authors, without undue reservation.

## Ethics Statement

The studies involving human participants were reviewed and approved by Hannover Medical School Ethics Committee. The patients/participants provided their written informed consent to participate in this study.

## Author Contributions

FC and PG were responsible for statistical analysis, data interpretation, and drafting the manuscript. MH was responsible for study design and implementation of the study. KO and HG was responsible for study design, implementation of the study, and data collection. JF and HAG were responsible for implementation of the study and data collection. TM was responsible for study design, implementation of the study, data collection, and conducting the interviews. JK and MR were responsible for implementation of the study. IH was responsible for study design, statistical analysis, and data interpretation. KK was responsible for study design, implementation of the study, statistical analysis, and data interpretation. All authors contributed to the article, revised the manuscript and approved the submitted version.

## Conflict of Interest

KO has received fees for lectures and/or consultations from Actelion, Janssen, MSD, Bayer, United Therapeutics, GSK, Janssen, Pfizer, and Acceleron, all outside the present study. HAG has received personal fees from Actelion, personal fees from AstraZeneca, personal fees from Bayer, personal fees from BMS, personal fees from GSK, personal fees from Janssen-Cilag, personal fees from Lilly, personal fees from MSD, personal fees from Novartis, personal fees from OMT, personal fees from Pfizer, and personal fees from United Therapeutics, outside the submitted work. HAG has received fees from Actelion, Bayer, Gilead, GSK, MSD, Pfizer, and United Therapeutics, outside the present work. D-HP has received honoraria for lectures from Janssen-Cilag. MH has received honoraria for lectures and/or consultations from Acceleron, Actelion, Bayer, GSK, Janssen, MSD, and Pfizer, all outside the present study. KK has received honoraria for consultations and/or lectures from Eli Lilly, Janssen, Lundbeck, Neuraxpharm, Otsuka, Pfizer, Servier, Schwabe, Takeda, and Trommsdorff/Ferrer, Alexio and CannaXan (advisory board). The remaining authors declare that the research was conducted in the absence of any commercial or financial relationships that could be construed as a potential conflict of interest.

## Publisher's Note

All claims expressed in this article are solely those of the authors and do not necessarily represent those of their affiliated organizations, or those of the publisher, the editors and the reviewers. Any product that may be evaluated in this article, or claim that may be made by its manufacturer, is not guaranteed or endorsed by the publisher.
